# Comparing the diagnostic value of Echocardiography In Stroke (CEIS) – results of a prospective observatory cohort study

**DOI:** 10.1186/s12883-021-02136-5

**Published:** 2021-03-17

**Authors:** Marlena Schnieder, Mohammed Chebbok, Michael Didié, Frieder Wolf, Mostafa Badr, Ibrahim Allam, Mathias Bähr, Gerd Hasenfuß, Jan Liman, Marco Robin Schroeter

**Affiliations:** 1 Department for Cardiology & Pneumology/Heart Center, University Medical Center Göttingen, Robert-Koch Straße 40, 37075 Göttingen, Germany; 2 Department for Neurology, University Medical Center Göttingen, Robert-Koch Straße 40, 37075 Göttingen, Germany

**Keywords:** Stroke, Echocardiography, Patent foramen ovale, Cardio-embolic stroke

## Abstract

**Background:**

Echocardiography is one of the main diagnostic tools for the diagnostic workup of stroke and is already well integrated into the clinical workup. However, the value of transthoracic vs. transesophageal echocardiography (TTE/TEE) in stroke patients is still a matter of debate. Aim of this study was to characterize relevant findings of TTE and TEE in the management of stroke patients and to correlate them with subsequent clinical decisions and therapies.

**Methods:**

We evaluated *n* = 107 patients admitted with an ischemic stroke or transient ischemic attack to our stroke unit of our university medical center. They underwent TTE and TEE examination by different blinded investigators.

**Results:**

Major cardiac risk factors were found in 8 of 98 (8.2%) patients and minor cardiac risk factors for stroke were found in 108 cases. We found a change in therapeutic regime after TTE or TEE in 22 (22.5%) cases, in 5 (5%) cases TEE leads to the change of therapeutic regime, in 4 (4%) TTE and in 13 cases (13.3%) TTE and TEE lead to the same change in therapeutic regime. The major therapy change was the indication to close a patent foramen ovale (PFO) in 9 (9.2%) patients with TTE and in 10 (10.2%) patients with TEE (*p* = 1.000).

**Conclusion:**

Major finding with clinical impact on therapy change is the detection of PFO. But for the detection of PFO, TTE is non inferior to TEE, implicating that TTE serves as a good screening tool for detection of PFO, especially in young age patients.

**Trial registration:**

The trial was registered and approved prior to inclusion by our local ethics committee (1/3/17).

## Background

About 20–40% of ischemic strokes are of cardioembolic source [1]. Atrial fibrillation is one major cardiac risk factor, besides several others such a patent foramen ovale (PFO), intracardial thrombi, aortic plaques or valvular disease [2]. Cardiac and aortic sources of emboli such as intracardial thrombi and aortic plaques are better visualized in transesophageal echocardiography (TEE), which is more cost intensive and semi-invasive, than in transthoracic echocardiography (TTE) [3] . Additionally there is also evidence for a higher sensitivity of TEE detecting cardioembolic sources of embolic stroke compared to TTE [4]. Moreover TEE is also believed to be superior in the workup of the diagnostic for PFO [3]. Recommendations are based on earlier studies, which showed a higher detection rate of PFO in TEE [5]. Despite being less specific transcranial doppler provided a higher detection rate of right-left shunts in comparison to TTE [6]. Without application of contrast agent and potential underdetection of PFO a subgroup analysis of the NAVIGATE-ESUS trial, TEE was more potent in detection of PFO than TTE [7]. Moreover, recent meta-analysis could demonstrate that TEE was superior to TTE in detecting intracardial abnormalities. This raises the question of the impact on change in therapy [8]. This prospective study aims to compare the diagnostic relevance of TEE to TTE in the perspective of additional abnormal findings and with an emphasis on clinical relevance for implication on change in therapeutical regime.

## Methods

From November 2017 till October 2019 patients admitted to our stroke unit or neurological intensive care unit of our university medical center with a transient ischemic attack or ischemic stroke were prospectively enrolled in this study. All patients eligible for the study underwent a standard diagnostic work up including a 12-lead electrocardiogram (ECG), holter-ECG with a duration of 72 h, duplex sonography of the head and neck arteries, a routine blood examination including HbA1c and the lipid status as well as infections parameters and creatinine. Inclusion criteria were age over 18 and patients had to be able to give capacity for consent. Exclusion criteria were a body mass index > 35 kg/m2, due to the reduced echocardiographic ability in obese patients and therefore per se decreased diagnostic value of TTE; as well as a mechanical heart valve and a suspected endocarditis would be a mandatory implication for TEE. The trial was registered and approved prior to inclusion by our local ethics committee (1/3/17), informed consent was obtained from all patients. Neurological characteristics, such as the etiology of the stroke, National Institute of Health Score (NIHSS) and modified Rankin Scale (mRS) were collected as well as the cardiovascular risk factors (CVRF) and potential anticoagulation or antithrombotic drugs. Each patient underwent subsequently transesophageal and transthoracic echocardiography following a predefined protocol by different experienced echocardiologists (F.W, M.C and M.D). They were blinded to patients imaging and clinical data, especially regarding echocardiography results. To minimize/optimize interrater variability/reliability the cardiologist analyzed 25 different predefined echocardiography recordings regarding major echocardiographic findings prior to the enrollment of study patients. Transesophageal and transthoracic echocardiography (TEE/ TTE) with IE33, CX50 and X7-2t probe (Philips Medical Systems, Eindhoven, Netherlands) or Vivid E9 and 6VT-Dprobe (GE Healthcare, USA) was performed by experienced cardiological examiners using a standard operating procedure. The collected data included the presence of a thrombus in the left atrial appendage (LAA), left atrium (LA) or the left ventricle (LV), measurements of LAA flow velocity, the LA dimension, the ejection fraction of the left ventricle as well as the presence of a PFO or a PFO combined with an atrial septum aneurysm (ASA), myxoma, aortic thrombus or plaque, the examination of heart valves including presence of calcifications or suspected endocarditis. The size of the PFO was defined by the number of bubbles passing in the left atrium. < 6 bubbles were defined as small, 6–26 as medium and > 26 as large PFO. Echocardiographic risk findings were divided in major and minor cardiac risk factors of cardio embolic stroke [9]. Descriptive statistics were presented using mean and standard deviation. Comparisons between detections rates in TTE and TEE were made using McNemar’s test. Groups comparisons between categorical variables were made using chi-squared test or Fischer’s exact test. With a *p*-value < 0.05 results were considered at statistically significant. Statistical analysis was performed using IBM SPSS Statistics vs 26.

## Results

**Fig. 1:**
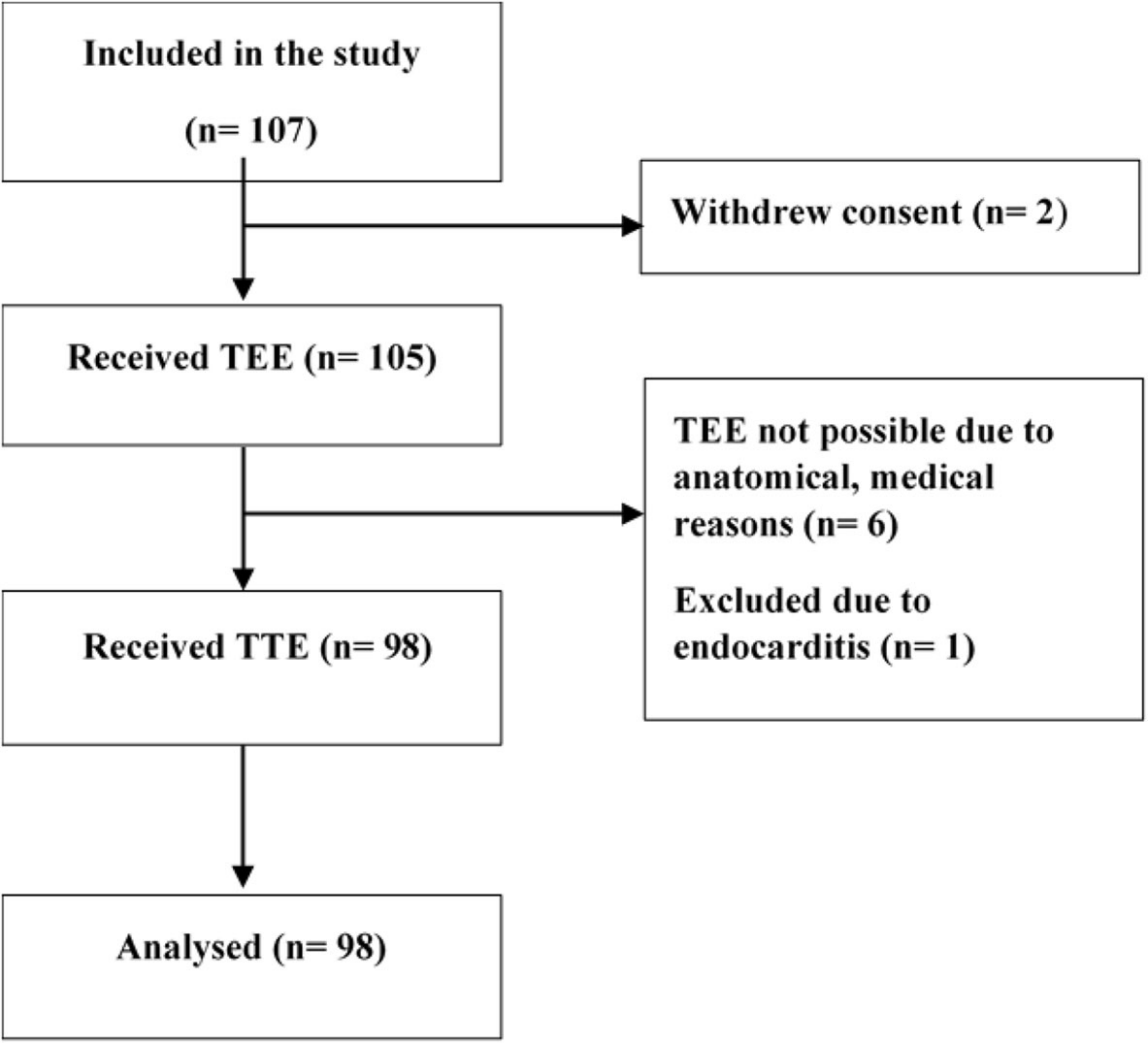
Flowchart of the patient selection

From November 2017 till October 2019 we enrolled 107 patients in our study. In this timespan 98 of 107 patients received TEE and TTE. Two patients withdrew consent. One patient was excluded due to a possible endocarditis. Due to anatomical reasons, pharyngeal bleeding or strong retching TEE was not possible in 6 patients (Fig. 1).

Baseline characteristics and neurological characteristics are listed in Table 1. 30 (30.6%) patients were female, 68 (60.4%) were male. Arterial hypertension was the most common CVRF with 62.2% (61 patients). Moreover, 55 (56.1%) suffered from dyslipidemia and 13 (13.3%) from diabetes. Atrial fibrillation and coronary heart disease were present in 10 (10.2%) patients respectively. The major etiology of stroke showed to be cardio-embolic in 26 patients (26.5%), while the localization was predominantly in the MCA territory (*n* = 66; 67.3%). The NIHSS as well as mRS at admission were rather low with in mean 2.7 (± 3.9) and 1.2 (± 1.2).

**Table 1:** baseline and neurological characteristics

baseline characteristics	
female	30 (30.6%)
age (years)	63.6 (± 12.5)
body mass index	26.3 (± 4.1)
smoking	28 (28.6%)
arterial hypertension	61 (62.2%)
dyslipidemia	55 (56.1%)
diabetes mellitus	13 (13.3%)
atrial fibrillation	10 (10.2%)
coronary heart disease	10 (10.2%)
heart failure	11 (11.2%)
thrombophilia	1 (1%)
**neurological characteristics**	
cardio-embolic	26 (26.5%)
macroangiopathy	9 (9.2%)
microangiopathy	20 (20.4%)
ESUS	11 (11.2%)
cryptogenic	18 (18.4%)
other etiology	14 (14.3%)
NIHSS at admission	2.7 (± 3.9)
mRS at admission	1.2 (± 1.2)
**territory**	
MCA	66 (67.3%)
ACA	1 (1%)
PCA	10 (10.2%)
BA	19 (19.4%)
systemic thrombolysis	4 (4.1%)
mechanical thrombectomy	9 (9.2%)

± = ± standard deviation, *ESUS* embolic stroke of unknown source, *NIHSS* National Institute of Health Stroke Score, *mRS* modified Rankin Scale, *MCA* middle cerebral arteries, *ACA* anterior cerebral arteries, *PCA* posterior cerebral arteries, *BA* basilar artery

Potential cardiac risk factors detected by echocardiography were found in 61 (62%) patients. Several of them had multiple findings, which together showed 116 times potential embolic risk factors. Major cardiac risk factors were found in 8 of 98 (8.2%) patients, 4 in TTE and 5 in TEE (Table 2). One myxoma and two fibroelastoma were found as major cardic risk factors. One patient with fibroelastoma was referred to a cardiac valve operation; the other received antithrombotic medication and a follow-up TEE after 6 months due to the small size of the fibroelastoma. The patient with the myxoma received oral anticoagulation, since further treatment was not desired by the patient.

**Table 2:** Major cardiac risk factors found by echocardiography

Major cardiac risk factor	TTE	TEE	p-value
LV-EF < 30%	2 (2%)	n.a.	n.a
LA thrombus	none	none	n.a
LAA thrombus	none	none	n.a
LV thrombus	1 (1%)	none	n.a
Mitral valve stenosis	1 (1%)	1 (1%)	n.a
Atrial myxoma	none	1 (1%)	n.a
Infective endocarditis	none	1 (1%)	n.a
Cardiac fibroma	none	2 (2%)	n.a

*TTE* transthoracic echocardiography, *TEE* transesophageal echocardiography, *LV-EF* left ventricular ejection fraction, *LA* left atrium, *LAA* left atrial appendage, *LV* left ventricle, *n.a.* not applicable

Due to the low number of findings in each major cardiac risk factor, no information about statistical significance of differences in TTE and TEE could be obtained. Minor cardiac risk factors for stroke were found in 108 cases, of whom 41 were detected via TTE and 105 via TEE (Table 3).

**Table 3:** Minor cardiac risk factors found by echocardiography

Minor cardiac risk factor	TTE	TEE	p-value
Mitral valve prolapse	1(1%)	9 (9.2%)	0.008
Mitral annular calcification	5 (5.1%)	27 (27.6%)	< 0.001
Calcified aortic stenosis	6 (6.1%)	5 (5.1%)	1.000
Patent foramen ovale	19 (19.4%)	18 (18.4%)	1.000
Spontaneous echo contrast	2 (2%)	5 (5.1%)	0.375
Atrial septum aneurysm	6 (6.1%)	11 (11.2%)	0.180
Left ventricular aneurysm	2 (2%)	none	n.a.
Aortic plaque	none	30 (30.6%)	< 0.001

*TTE* transthoracic echocardiography, *TEE* transesophageal echocardiography, *n.a.* not applicable

PFO was detected in 18 (18.4%) patients by TEE and 19 (19.4%) by TTE. One false positive result occurred in TEE group. After further diagnostics it appeared as a pulmonary right-left shunt. We did find significant more mitral-valve prolapses in TEE comparing to TTE (9 (9.2% vs. 1 (1%); *p* = 0.004) as well as annular calcifications (27 (27.6%) vs. 5 (5.1%); *p* < 0.001) and aortic plaques (30 (30.6%) vs. 0 (0%); p < 0.001). Looking closer at the clinical relevance of those echocardiographic findings, we saw an overall change in therapeutic regime by TTE and/or TEE in 22 (22.5%) cases. In 5 (5%) cases of TEE, in 4 (4%) TTE and in 13 cases (13.3%) TTE and TEE lead to a change in therapeutic regime. Stroke relevant therapeutic changes were present in 13 (13.2%) cases, while other findings were cardiac related therapy changes (Table 4).

**Table 4:** Impact of echocardiography on therapy change

therapy change	TTE	TEE	p-value
**stroke related therapy changes**			
closure of patent foramen ovale	9 (9.2%)	10 (10.2%)	1.000
antibiotic therapy	none	1 (1%)	n.a.
oral anticoagulation	none	2 (2%)	n.a.
**cardiac related therapy change**			
heart failure therapy	2 (2%)	1 (1%)	n.a.
coronary angiography	5 (5.1%)	3 (3.1%)	0.500
coronary stent	1 (1%)	none	n.a.
heart valve operation	1 (1%)	1 (1%)	n.a.
coronary artery bypass graft	2 (2%)	1 (1%)	n.a.
dual antiaggregation therapy	1 (1%)	none	n.a.
other	4 (4.1%)	4 (4.1%)	1.000

*TTE* transthoracic echocardiography, *TEE* transesophageal echocardiography, *n.a.* not applicable

The major change was the indication of PFO-closure in 9 (9.2%) patients with TTE and in 10 (10.2%) patients with TEE (p = 1.000). Regarding the size of the PFO, there was slight difference in detection. In TTE, two showed to be small, two moderate and 14 large sized, while in in TEE, one was small, six moderate and eleven large sized (*p* = 0.619). Frequent findings of other minor cardiac risk factors, which were more likely to be detected in TEE such as aortic plaque, annular mitral valve calcifications or mitral valve prolapse did not lead to a change in therapeutic regime. Since TEE is especially recommended in younger patients with years of age under 61, we analyzed for possible differences in detection rates depending on the age of the patients. In total 42 patients were under 61 years. In this group major cardiac risk factors were rare and found in 7.1% of patients (Table 5).

**Table 5:** Echocardiographic findings in patients ≤60 years

Cardiac risk factors in patients ≤ 60 years	TTE	TEE	p-value
**major cardiac risk factors**			
LV-EF < 30%	1 (2.4%)	n.a.	n.a
LA thrombus	none	none	n.a
LAA thrombus	none	none	n.a
LV thrombus	none	none	n.a
mitral valve stenosis	none	none	n.a
atrial myxoma	none	1 (2.4%)	n.a
infective endocarditis	none	none	n.a
cardiac fibroma	none	1 (2.4%)	n.a
**Minor cardiac risk factors**			
mitral valve prolapse	none	4 (9.5%)	*p* = 0.125
mitral annular calcification	1(2.4%)	4 (9.5%)	p = 0.250
calcified aortic stenosis	none	none	n.a.
patent foramen ovale	13 (31%)	12 (28.6%)	p = 1.000
spontaneous echo contrast	none	none	n.a.
atrial septum aneurysm	4 (9.5%)	7 (16.7%)	p = 0.375
left ventricular aneurysm	none	none	n.a.
aortic plaque	none	6 (14.25%)	p = 0.031

*TTE* transthoracic echocardiography, *TEE* transesophageal echocardiography, *LV-EF* left ventricular ejection fraction, *LA* left atrium, *LAA* left atrial appendage, *LV* left ventricle, *n.a.* not applicable

In TEE, two patients with fibroelastoma were detected and in TTE one patient had a reduced left ventricular ejection fraction under 30%. Furthermore, no other major cardiac risk factors were found in TTE nor in TEE. Regarding minor cardiac risk factors, PFO is the most frequent finding in TEE 12/42 (28.6%) and TTE 13/42 (31%) (*p* = 1.0). Other risk factors such as atrial septum aneurism were shown in 7/42 (16.7%) in TEE and 4/42 (9.5%) in TTE (*p* = 0.375). Spontaneous echocontrast was found in 1/42 (2.4%) in TEE and TTE, aortic plaques in 6/42 (14.25%) in TEE and none in TTE (*p* = 0.031), annular mitral valve sclerosis in 4/42 (9.5%) in TEE and in 1/42 (2.4%) in TTE (*p* = 0.250). Mitral valve prolapse was found in 4/42 (9.5%) in TEE and none in TTE (*p* = 0.125). Other minor cardiac risk factors such as LV aneurysm or aortic stenosis were not found in TEE or TTE. Comparing the findings of younger (≤ 60 years) to older patients, there were a higher rate of PFO (12 (28.6%) vs. 6 (10.7%); *p* = 0.034) in younger patients and higher rate of aortic plaque (6 (14.3%) vs. 24 (44.4%); *p* = 0.002) and mitral annular calcifications (4 (9.5%) vs. 23 (41.1%); *p* = 0.001) in older patients. In 14/42 (33.3%) of younger patients, echocardiography lead to a therapy change. This was mainly the detection respectively closure of PFO in 8/42 (19%) patients after TEE and in 7/42 (16.7%) patients after TTE (*p* = 1.000). One patient (2.4%) received oral anticoagulation after TEE and none after TTE. Other therapy changes were related to cardiac therapy and not stroke relevant. One patient (2.4%) received dual platelet inhibition after TTE and none after TEE. The same hold true for coronary angiography, coronary artery bypass graft and implantation of cardiac stent. Two out of 42 (4.8%) patients received heart failure therapy after TTE and One (2.4%) after TEE.

## Discussion

Echocardiography revealed abnormal findings in 62% of patients, which in 22.5% lead to a change in therapeutic regime. TEE does give additional information with clinical relevance regarding detection of the etiology of stroke, but the number of abnormal findings leading to a change in the therapeutical regime, which were not primally detected in TTE is rather low (5%). On the other hand, TTE revealed exclusive findings in stroke patients compared to TEE, e.g. reduced LV-EF and need of heart failure therapy or thrombi in the left ventricle. In our study major cardiac risk factors were found in 8.2% by TEE, especially left atrial thrombi were rare. Patients with sinus rhythm were infrequently detected with left atrial thrombi [10], therefore emphasis in clinical routine should be put on rhythm monitoring. Overall the prevalence of major cardiac risk factors was lower than in other studies [11], but we did not differentiate aortic plaques regarding their size and complexity in major and minor cardiac risk factors. Minor cardiac risk factors such as mitral valve annular calcifications or prolapse of the mitral valve were more frequent to be found in TEE than in TTE due to the better view on heart valves in TEE because their closer proximity of the imaging transducer ^12^. But looking closer at those findings, they did not have any impact on the therapeutical regime. The most common finding in TEE was the plaque in the aortic arche. An aortic plaque > 4 mm is an independent risk factor of recurred stroke [12] and well detectable in TEE but not in TTE. However, it has been shown that a normal carotid intima-media-thickness has a negative predictive value for aortic plaques [13] and computed tomographic angiography has a negative predictive value for high grade aortic arche atheroma [14]. Furthermore, a therapy change after detection of aortic plaque is doubtful, since patients receive antithrombotic drugs after a stroke as part of the regular therapeutic regime or even, after a minor stroke or high-risk TIA, a dual platelet inhibition [14, 15]. Diagnostics of patent foramen ovale came more into focus since successful studies demonstrated the benefit of closure of PFO in younger patients with cryptogenic stroke [16–18]. Previous studies showed, that TEE is less sensitive in detection of PFO than transcranial doppler [6] and that the detection rate is lower in TTE than in TEE [5]. In contrast, our study revealed that TTE serves as a good screening tool for detection of PFO, since the detection rate is similar to TEE. Only the precision in the determination of the size of the patent foramen ovale seems to be better in TEE than in TTE, which has also been shown before [19]. In the subgroup analysis of the younger patients aged ≤60 years, we found a higher rate of PFO with 28.6% as expected, but the detection rate of PFO was similar in TTE and TEE in this group. Only three major cardiac risk factors found in TEE in patients age ≤ 60 years, leading to the suggestion/discussion if TEE should not be routinely performed in younger patients but rather could be reasonable in older stroke patients as suggested before [11] or dispensable under certain conditions, since the detection rate of PFO could be similar in TTE as in TEE. We excluded patients with clinically suspected endocarditis in our study, since TEE would be the method of choice in suspected endocarditis due to its higher sensitivity and specificity in diagnosis of endocarditis than TTE [20]. The aim of our study was not to examine the relevance of TEE in patients with a clear indication for it, but rather test its added diagnostic value compared to TTE in patients without classical indications for this procedure.

## Conclusions

Major finding of echocardiography in stroke patients with clinical impact on therapy change is the detection of a relevant PFO. In this matter, TTE is equally sufficient to TEE, implicating a good screening tool for detection of PFO in cryptogenic stroke, especially in younger patients ≤60 years. However, TEE remains the gold standard in specific situations or indications, such as endocarditis, obese patients and rare causes of embolic stroke (e.g. myxoma and fibroelastoma).

## Data Availability

The datasets generated and analysed during the current study are not publicly available due to the possibility of compromising individual privacy but are available from the corresponding author on reasonable request.

## Abbreviations

TTE: Transthoracic echocardiography
TEE: Transesophageal echocardiography
PFO: Patent foramen ovale
ECG: Electrocardiogram
NIHSS: National Institute of Health Stroke Score
mRS: modified Rankin Scale
CVRF: Cardiovascular risk factors
LAA: Left atrial appendage
LA: Left atrium
LV: Left ventricle
ASA: Atrial septum aneurysm
ESUS: Embolic stroke of unknown source
ACA: Anterior cerebral artery
MCA: Middle cerebral artery
PCA: Posterior cerebral artery
BA: Basilar artery
LV-EF: Left ventricular ejection fraction
